# Connected health for growth hormone treatment research and clinical practice: learnings from different sources of real-world evidence (RWE)—large electronically collected datasets, surveillance studies and individual patients’ cases

**DOI:** 10.1186/s12911-021-01491-0

**Published:** 2021-04-26

**Authors:** Nea Boman, Luis Fernandez-Luque, Ekaterina Koledova, Marketta Kause, Risto Lapatto

**Affiliations:** 1 Paediatric Endocrinology, Children’s Hospital, University of Helsinki and Helsinki University Central Hospital, Stenbackinkatu 11, PO BOX 281, 00029 Helsinki, Finland; 2 Adhera Health Inc., Palo Alto, CA USA; 3 Global Medical Affairs Cardiometabolic and Endocrinology, Merck KGaA, Darmstadt, Germany; 4 Medical Department, Merck Oy Finland (an affiliate of Merck KGaA, Darmstadt, Germany), Espoo, Finland

**Keywords:** EHealth, Adherence monitoring, Growth outcomes, Observational study

## Abstract

**Background:**

A range of factors can reduce the effectiveness of treatment prescribed for the long-term management of chronic health conditions, such as growth disorders. In particular, prescription medications may not achieve the positive outcomes expected because approximately half of patients adhere poorly to the prescribed treatment regimen.

**Methods:**

Adherence to treatment has previously been assessed using relatively unreliable subjective methods, such as patient self-reporting during clinical follow-up, or counting prescriptions filled or vials returned by patients. Here, we report on a new approach, the use of electronically recorded objective evidence of date, time, and dose taken which was obtained through a comprehensive eHealth ecosystem, based around the easypod™ electromechanical auto-injection device and web-based connect software. The benefits of this eHealth approach are also illustrated here by two case studies, selected from the Finnish cohort of the easypod™ Connect Observational Study (ECOS), a 5-year, open-label, observational study that enrolled children from 24 countries who were being treated with growth hormone (GH) via the auto-injection device.

**Results:**

Analyses of data from 9314 records from the easypod™ connect database showed that, at each time point studied, a significantly greater proportion of female patients had high adherence (≥ 85%) than male patients (2849/3867 [74%] vs 3879/5447 [71%]; *P* < 0.001). Furthermore, more of the younger patients (< 10 years for girls, < 12 years for boys) were in the high adherence range (*P* < 0.001). However, recursive partitioning of data from ECOS identified subgroups with lower adherence to GH treatment ‒ children who performed the majority of injections themselves at an early age (~ 8 years) and teenagers starting treatment aged ≥ 14 years.

**Conclusions:**

The data and case studies presented herein illustrate the importance of adherence to GH therapy and how good growth outcomes can be achieved by following treatment as described. They also show how the device, software, and database ecosystem can complement normal clinical follow-up by providing HCPs with reliable information about patient adherence between visits and also providing researchers with real-world evidence of adherence and growth outcomes across a large population of patients with growth disorders treated with GH via the easypod™ device.

## Background

A wide range of elements can affect health outcomes in many chronic conditions: individual response to treatment, adherence to treatment, environmental factors (e.g. nutrition), and other clinical aspects [1]. The treatment of these chronic conditions often necessitates long-term use of prescribed medications. Although such medications may be effective in ameliorating or suppressing symptoms and may even be able to halt or reverse disease progression, these positive outcomes are often not realized because approximately half of patients do not take their medications as prescribed [1–3].

Non-adherence to treatment can range from taking a smaller dose than prescribed to missing a dose intermittently to not taking the medication at all, and may be deliberate or due to forgetfulness or lack of understanding of the need to maintain adherence. The reasons for poor adherence are manifold. They include patient-related factors such as suboptimal health literacy, misplaced fears and beliefs about the medication, the discomfort of injections, and lack of involvement in the treatment decision-making process [1, 4, 5]. Healthcare provider (HCP)-related factors such as overly complex drug regimens, the inconvenience of injectable medications, ineffective communication with patients, inadequate discussion of the risk of adverse effects, difficulty in recognizing the risk factors for non-adherence, misinterpretation of the reasons for the reduced adherence, and lack of continuity of care can also contribute to poor adherence [1, 4, 6]. Even healthcare system-related factors, such as restrictions on consultation time, limited access to care due to cost or geography, and lack of health-related information technology may be involved. Because these potential barriers to good adherence to treatment can be so complex and varied, a range of different methods, technologies, and behavioral techniques may be required to overcome them [7].

One example of the importance of adherence is the management of growth disorders, where long-term adherence to growth hormone (GH) treatment, which can necessitate daily injections over many years, has been shown to determine the efficacy of the treatment [8, 9]. Traditionally, assessment of adherence to treatment has been based on subjective, relatively unreliable methods, such as patient self-reporting during clinical follow-up, or counting prescriptions filled or vials returned by patients [7, 10]. This has made it difficult for HCPs or researchers to accurately evaluate the role of adherence in an individual’s treatment response. More recently, however, advances in digital technology and mobile computing have enabled more reliable, accurate collection and sharing of patient data, such as number and timing of injections and dosage administered [11]. This has the potential to improve evaluation of adherence and patient behavior generally. Active engagement with the use of self-monitoring electronic devices has previously been shown to improve patients’ adherence to treatment in chronic conditions, such as diabetes and asthma, and is associated with the long-term improvement of clinical outcomes [11–15]. These innovative eHealth devices and technologies have the potential to improve health outcomes by seamlessly tracking adherence to the treatment plan, engaging and informing HCPs and, used with patient support programs (PSPs), providing greater insights to help the healthcare team to support their patients [11, 13, 14].

Electronic monitoring is now considered the gold standard for monitoring adherence,[16–18] and the easypod™ electromechanical delivery device (Merck KGaA, Darmstadt, Germany) is the first injection device able to provide objective prospective adherence data for GH therapy which can be recorded for subsequent analysis by the patient’s HCP team. The device has a front screen to display settings, data, and even a pictorial ‘How to use’ sequence. The prescribed dose, date, time, patient’s name, and language can be set and changed as required. Four adjustable comfort settings are available for needle speed, injection speed, injection depth, and injection duration. The date, time, and dose administered are recorded. The injection process is automatic and is triggered by pressing a button at the top of the device. The device can detect and advise the user when only a partial dose is left in one of the replaceable cartridges of GH and correct for this when a new cartridge is inserted. The date, time, and dose administered can all be transmitted by infrared to the docking station for the device, which can then transmit it using 3G telephony to the patient’s HCP and to the easypod™ database on a secure server via easypod™ connect web-based software for analysis.

The real-time electronic monitoring enabled by this device has the potential to benefit both the patient and the HCP, as it may provide a starting point for discussions on any issues identified from the data, such as an individual patient failing to maintain adherence over time. Electronic monitoring illustrates how accurate monitoring of an individual’s injection pattern can not only track overall adherence, it can also identify specific occasions when non-adherent behaviors occur, enabling the HCP involved to directly intervene and initiate a personal discussion with the patient. This can help to motivate and enable the patient to make positive behavioral changes to improve adherence and therefore obtain optimal growth outcomes from their GH treatment. Monitoring adherence is also valuable in identifying whether an individual’s sub-optimal growth is due to low adherence or to a lack of treatment efficacy [18, 19]. The output can be used proactively on a population level, to identify age groups or periods when patients may need particular attention. For example, the onset of puberty frequently results in a drop in adherence, particularly when patients transition from a regimen overseen by parents or caregivers to self-administration [20, 21].

The data collected by the device include not only the date and time a dose is taken, and the dose administered, but also the comfort settings used (e.g. speed of injection, depth of needle). Other types of real-world data (RWD) that could be collected and used, complementary to the data automatically collected by the device, are observational data generated in routine clinical practice, beyond the scope of randomized controlled trials (RCTs) [22]. These include health-related data reported and collected in real-world medical settings, from electronic health records (EHRs), claims databases, health surveys, patient registries, or obtained directly from health-related apps, mobile devices, and social media. In turn, RWD can be utilized, through the use of computing power and interconnectivity, to generate real-world evidence (RWE).

In this paper, we aim to provide an overview of a complete GH treatment eHealth platform and describe how it has been used to create one of the largest longitudinal and observational databases currently available, as part of a comprehensive eHealth ecosystem. To illustrate the value of this ecosystem, we present two patient case studies as examples of how electronic monitoring can be used to provide educational information and evidence that is of potential clinical relevance.

## Methods

**Fig. 1:**
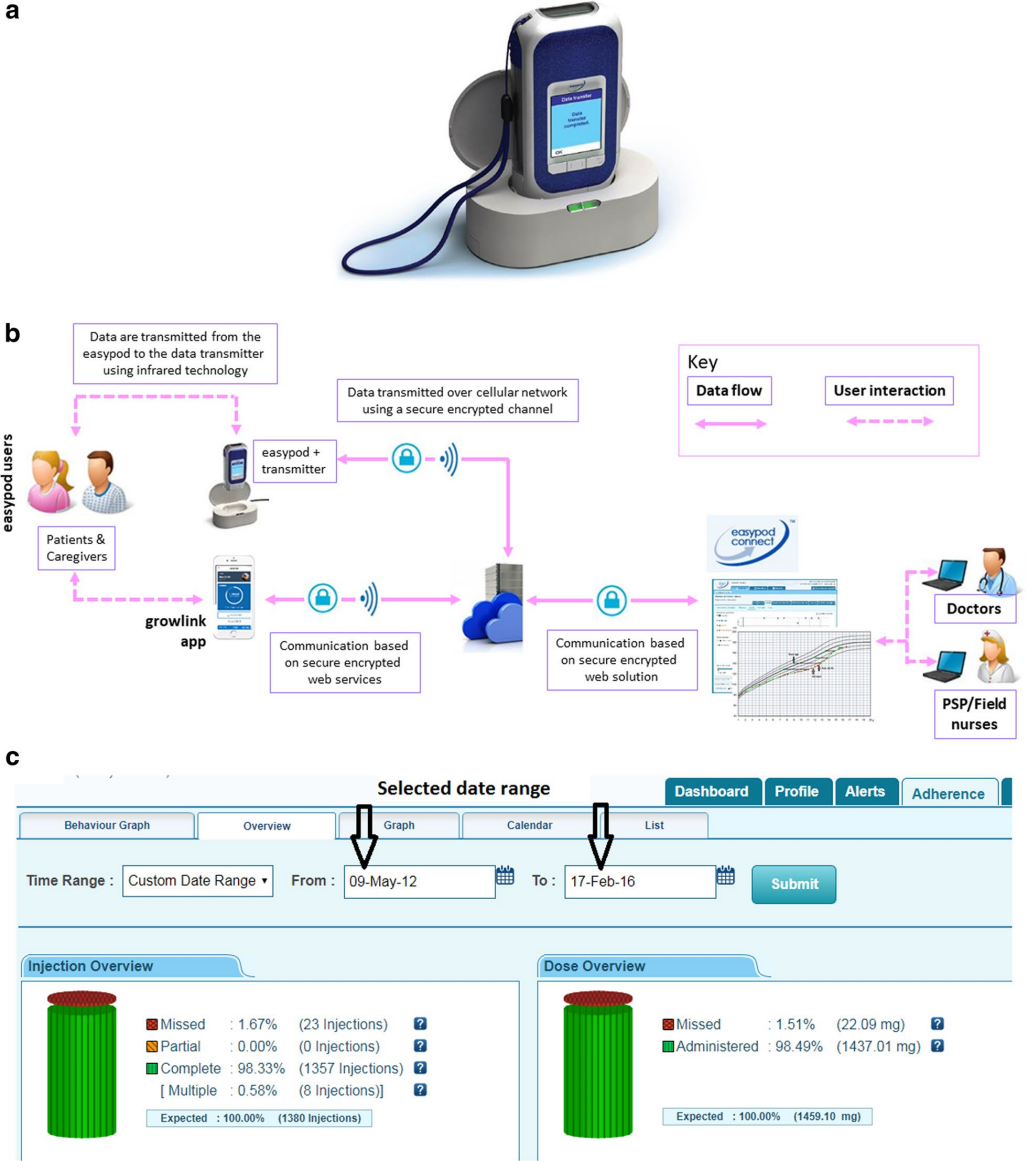
The easypod™ autoinjection smart device and the transmitter and web interface of the ecosystem. **a** The easypod™ autoinjection device and the transmitter that allows the transmission of data to easypod™ connect for database storage and analysis. **b** The complete easypod™ ecosystem. **c** A screenshot from the web interface showing injection and dose history overview. Image reprinted with permission of Merck KGaA, Darmstadt, Germany

This eHealth ecosystem is based upon the easypod™, a digitally enabled electromechanical GH injection device currently approved for use in more than 40 countries, including the EU and the USA [23]. The ecosystem also includes Connect web-based software [24], which stores the data in a secure database, and growlink™ [25], a recently developed mobile app which allows both HCPs and patient/caregivers to monitor a patient’s adherence to their treatment regimen (Fig. 1). The database incorporates data from the easypod™ Connect Observational Study (ECOS), a large, 5-year multinational study, [23] along with data from a range of other sources from 2007 to the present, including small scale, local studies, and individual clinicians who have patients receiving GH via the easypod™ device.

The delivery device electronically records adherence data (completed and missed doses) for patients receiving GH (Saizen® [somatotropin], Merck KGaA, Darmstadt, Germany) to treat growth disorders. The device can then transmit reliable, accurate data on the time, date, and dose injected via the use of a data transmitter with an infrared interface which is activated by the patient (Fig. 1). The data are forwarded to a cloud-based solution via the cellular network. HCPs can then access their individual patient’s adherence data via the web-based eHealth Connect software platform, but these data are anonymized by removing all Personal Identifiable Information (PII) when added to the database, to maintain patient privacy. growlink™ is a patient facing mobile app that has been developed to improve patients’ engagement with their treatment by providing them with visibility of their historical treatment adherence, as well as educational and motivational content.

Three datasets are incorporated into the ecosystem: RWD and clinical details from ECOS and other clinical trials, collectively regarded as RWE [26, 27]. RWD is collected directly from the connected electronic injectors used by the patients and stored in secure cloud servers. Additional RWD such as growth outcomes are captured for individual patients by the HCPs.

ECOS, cited earlier, was a real-world open-label, observational, longitudinal study conducted between 2010 and 2016, enrolling children from 24 countries who were being treated with GH via the auto-injection device. A total of 1190 patients provided accurate, real-time data on adherence and growth outcomes which were analyzed and reported and then added to the database as anonymized RWD, with study investigators adding other clinical information about the patients. In order to allow statistical analysis of these patient data, the patients signed a user agreement before enrolling in the study. To ensure privacy, the data were anonymized by removing all PII and by aggregating the results into sets of 10 patients before adding them into the database. The core features of the RWE dataset are the large total population included, the multinational setting, and automated recording of adherence and injection settings, which are supported by basic patient characteristics, such as age, sex, and pubertal status, which are manually entered by the HCPs involved. In some analyses, nominal cut points for age at puberty were employed, which were 10 years for girls and 12 years for boys.

ECOS was conducted in accordance with the principles of the Declaration of Helsinki, Good Clinical Practice (ICH-GCP E6) guidelines, and applicable national legal and regulatory requirements. Written informed consent was obtained from patients (or their parent/guardian) prior to study enrolment.

Anonymized data from ECOS have been added into the RWD database to enable researchers to study adherence to GH therapy in different age groups, and to assess the effects of age and sex on adherence and growth outcomes and other aspects of GH treatment. Access to the database is, however, only available to individual HCP accounts for patients treated by that HCP, for PSPs, or as a part of a research study, in order to ensure scientific rigor and to maintain patient confidentiality.

Recursive partitioning, a mathematical modelling technique using the statistical software R (rpart^3^), with cross-validation was used in one sub-analysis of ECOS data to explore subgroups of children with relatively low and high adherence in the first and second years on GH treatment [28]. Recursive partitioning is the step-by-step process in which a decision tree is constructed by either splitting or not splitting each variable on the tree into two subgroups. The method splits the total group into two subgroups according to a cut-off value, for example, for age. Then further splits can be made within each subgroup. The algorithm considers all possible splits for all possible variables and selects the variable and split that creates the most homogeneous subgroups. Children were selected from ECOS for this analysis if they had GH deficiency (GHD, n = 314), Turner syndrome (n = 27) and small for gestational age (SGA, n = 35), were naïve for GH (defined as never having previously received any form of GH treatment), had a known target height, and complete data on growth and adherence in the first and second years (n = 376).

Two anonymized individual patient case studies are also presented here, selected from the cohort of ECOS in Finland because of their different treatment experiences.

## Results

The data captured in the easypod™ ecosystem database currently include longitudinal records for 13,553 children transmitting data on > 10 injections from January 2007 to February 2019 [29]. The 10 injection cut-off point was imposed to exclude test or training injections from when therapy is initiated.

Analysis of results from the overall database [24] and separate analyses of data from ECOS [23] suggest that pubertal children, especially boys, are at greater risk for suboptimal adherence and may benefit from closer engagement, particularly around the age when they start to take responsibility for their injections [24, 28, 29]. Specifically, analyses of data from 9,314 records from the overall database showed that, at each time point studied, a significantly greater proportion of female patients had high adherence (defined as ≥ 85% [medium: > 56% to < 85%; low: ≤ 56%]) than male patients (2849 of 3867 [74%] vs 3879 of 5447 [71%]; *P* < 0.001) [24]. In addition, more of the younger, pre-pubertal patients (< 10-years-old for girls, < 12-years-old for boys) were in the high adherence range (*P* < 0.001). However, recursive partitioning, with adherence in the first year as an outcome, identified two subgroups with relatively low adherence to GH treatment. These were children who performed the majority of injections themselves and who were < 8 years of age at the start of treatment (35% mean adherence, n = 10) and children who performed the majority of injections themselves and who were ≥ 8 years of age and had a weight ≥ 0.5 standard deviation score (SDS), adjusted for age and sex at start of treatment (57% mean adherence, n = 22) [28]. Recursive partitioning with adherence in the second year as an outcome identified another group with relatively low adherence to GH treatment – teenagers aged ≥ 14 years when they started treatment (70% mean adherence) [28]. Early identification of subgroups of children with relatively low adherence to GH treatment through automated continuous assessment of adherence via easypod™ makes it possible for their HCPs to intervene to improve their adherence and subsequent growth outcomes.

Overall, however, global results from ECOS demonstrated clearly that use of the easypod™ device enabled young patients to maintain high levels of adherence over long periods of time. For example, over the first year of monitoring for the ECOS easypod™ adherence data analysis set (DAS; n = 1190), the median rate of adherence was 93.7% among patients overall and > 93.0% in GH-naïve patients, irrespective of the treatment indication. Clinically meaningful improvements in growth rates were observed after 1 year of treatment across the various GH indications involved, including GHD, Turner Syndrome, and SGA. Within the easypod™ adherence DAS, overall, the median change in height SDS from baseline to 1 year was 0.47 (Q1; Q3 0.27; 0.68) across each of the indication groups; median overall height velocity (HV) over the first year of treatment was 8.2 (6.9; 9.4) cm/year and HV SDS was 2.11 (0.60; 3.62), indicating a positive growth response [23]. The overall median adherence rate remained high at the end of 3 years of follow-up: 87.2% (n = 409), 75.5% after 4 years (n = 143), and 70.2% after 5 years (n = 43). ECOS also demonstrated statistically significant Spearman's product-moment correlations between adherence and 1-year change in height standard deviation score (0.11; p < 0.001 for patients overall) and height velocity (0.14; p < 0.001) [23]. Assessments of data from ECOS cohorts in a number of countries have demonstrated similar results, despite differences in the health systems in the various countries involved [30, 31].

The case studies presented here were selected from the ECOS cohort in Finland. Children and adolescents starting GH treatment in Finland have a reasonably high degree of physician and nurse contact at public clinics—initially every 3 months. The clinical trial data do not enable the identification of individual patterns of patient management, but these two individual patient cases provide more detailed examples of how interventions can be tailored according to day-to-day problems with adherence. Selection of patients with such different experiences of treatment may provide a valid starting point for a meaningful discussion on the most appropriate interventions to maintain adherence. The first patient had an early diagnosis and took over administering injections from the parents at the age of 9.5 years (Case study Patient A). The second patient was diagnosed later, aged 11 years, and self-administered injections from the start of treatment but with full parental support throughout the treatment period (Case study Patient B).

### Patient A

This patient had a diagnosis of idiopathic GHD but had no other relevant medical history or therapy, brain magnetic resonance imaging (MRI) was normal and HV was 1.9 cm/year. The patient started GH treatment when their height was − 2.8 SDS. The expected adult height for this patient, based on population height data and parental height, was + 0.4 SDS. Initially, the parents administered the injections with an adherence of 87%, but when nearly 10 years old, the patient took over and began self-injecting, and adherence dropped to 77%.

When the patient visited the clinic a year later with their mother, they were asked about adherence, and the patient admitted that they had missed a few injections each month since the last visit. However, it could be seen from the electronic injection record that the patient had missed a few injections every week since the spring period, and that the injections were mostly missed during the weekends. The HCP asked about the drop in adherence and reviewed the printed data together with the patient. It became apparent that the parents were not aware that the patient had missed so many injections. They had not checked the injection data regularly on the device’s calendar and had trusted their child’s word when the child said that they had taken their injections.

The patient had started wrestling as a hobby 8 months earlier, and had attended training camps during the weekends. The charts showed that adherence varied month by month, for example in May adherence was 61% but in August it was 94%. The patient explained that during the weekend wrestling training camps they did not want to take the GH injection because their friends would see that and there was also the inconvenience of storing the device and reconstituted cartridge in a refrigerator. Thus, there were more missed injections during the weekends and especially during the training camp months.

Together, the patient, the mother and the HCP came up with the following solutions: the HCP advised that GH (Saizen®) could be stored at room temperature (maximum 25 °C) for up to 7 days, removing the need for refrigeration when at the training camp. The patient could then take the injections in the privacy of the toilet/bathroom during the training camps without their friends knowing. The parents would check adherence from the device more often and also provide support at home to improve adherence between training camps. The HCP also emphasized to the patient the importance of adherence to get maximum benefit from the therapy and achieve the expected adult height. The HCP requested that the family should contact them as soon as possible if more problems occurred, so that together they could find solutions to ensure the clinical outcomes and the patient’s final adult height would not be affected. Due to early detection of decreased adherence there were no major changes to growth and other clinical parameters.

**Fig. 2:**
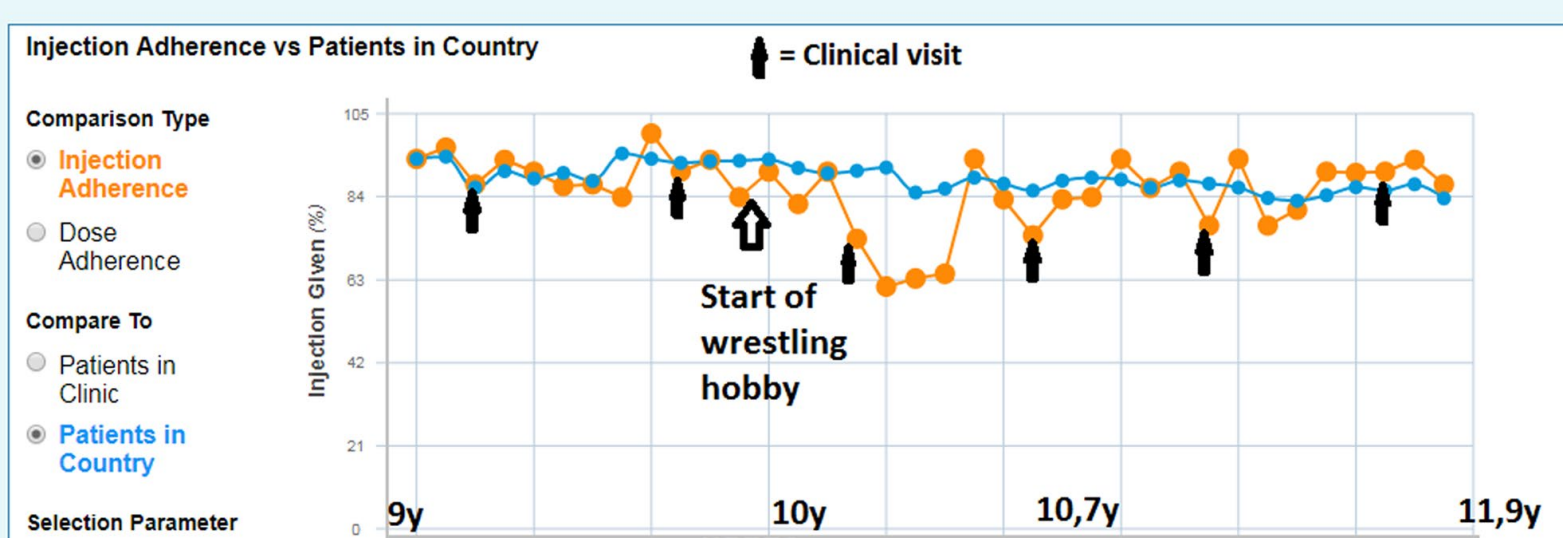
Injection adherence behavior graph for patient A

**Fig. 3:**
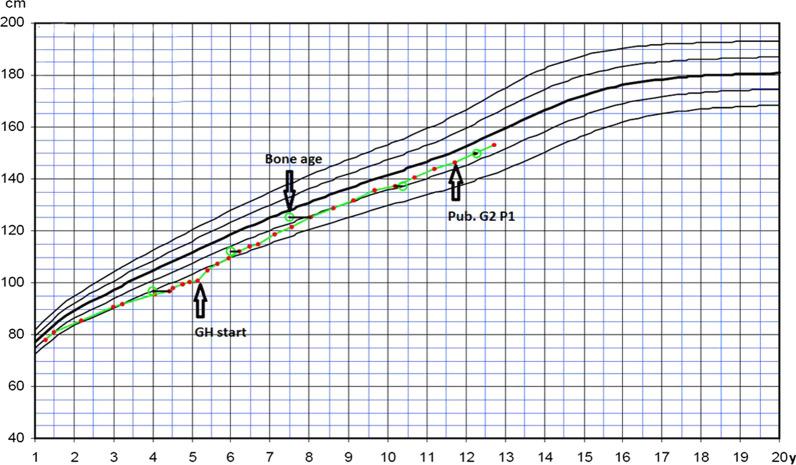
Growth chart for patient A

At a clinic visit at the age of 12 years it was noted that the patient’s adherence level was 87.05% and puberty had just started. The bone age was the same as the calendar age at 12 years and height SDS was − 0.6. There were no further variations in adherence between different months and seasons. Although the patient still missed some injections during the training camps or contest trips, they missed fewer than before (Fig. 2). GH-therapy will continue until the patient reaches adult height (Fig. 3).

### Patient B

This patient also had a diagnosis of GHD. The medical history included attention deficit hyperactivity disorder, gastrointestinal problems, anemia, and depression. The MRI was normal. The patient started GH treatment when they were 11 years old, height was − 2.8 SDS and their expected adult height was − 0.9 SDS. The GHD was diagnosed relatively late, which may have contributed to the other medical history. The patient self-administered the GH injections right from the start. Self-administration and the ability to observe dosage and growth progress gave the patient confidence and the parents’ assurance that injections were being adhered to. Puberty started at the age of 12.6 years.

**Fig. 4:**
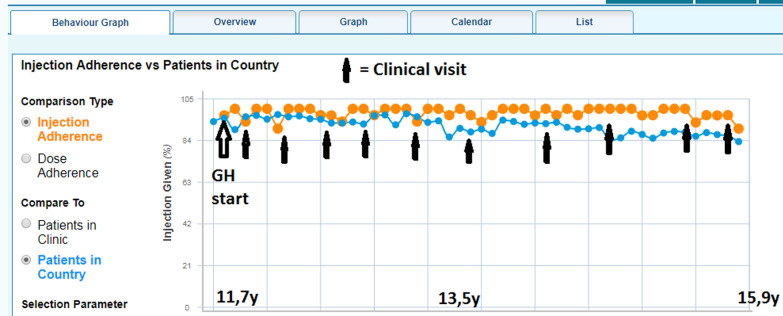
Injection adherence behavior graph for patient B

**Fig. 5:**
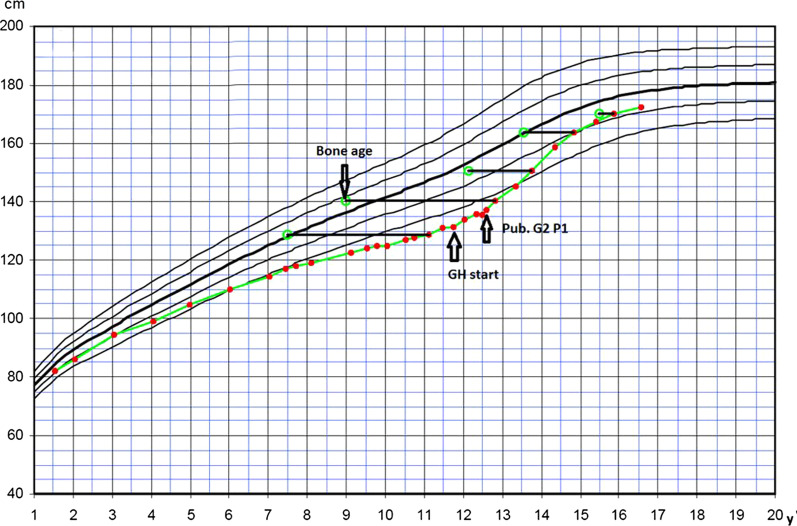
Growth chart for patient B

Over a 4-year treatment period, the patient’s adherence was extremely good at 98%. The patient was motivated to keep to the treatment schedule and maintained good adherence throughout this period. The patient wanted to catch up on the height of their friends and became more motivated at every clinical visit when they were measured. The patient’s HV had increased up to 13 cm/year during the treatment, demonstrating a good response. The patient and parents all understood that there was limited time left to grow in, so good adherence to GH treatment was very important to get maximum benefit and to reach the expected adult height despite starting treatment quite late. Although the patient self-administered the injections, the parents provided good support throughout the treatment period. They checked regularly to ensure that no injections had been missed. The patient’s pubertal development progressed without problems and did not affect adherence in any phase (Fig. 4). At a clinic visit when the patient’s bone age was 15.5 years, the calendar age was just under 16 years. The GH dose was decreased and it was decided that GH treatment could be stopped at the next clinic visit (Fig. 5).

## Discussion

There is great potential for learning in using RWE such as the large, long-term ECOS study and RWD such as those provided by the easypod™ device. Unlike RCTs, RWE studies avoid the over-selection of patients and the reinforcing effect on adherence (the Hawthorne effect) associated with taking part in a controlled trial. They can, however, carry a risk of bias if the protocol is not developed carefully enough. The RWD collected by patients from reliable, accurate devices such as glucometers and, as in the two case studies presented here, the easypod™ device, is considered to be a valuable resource for RWE, although RCTs continue to be regarded as the gold standard of medical evidence by many. The insights into real-world behavior and management of patients provided by RWE, however, cannot be obtained from RCTs and this is an example of how new knowledge and value can be derived directly from data.

Other large scale surveillance studies, for example, The Pfizer International Growth Database (also known as KIGS), the National Cooperative Growth Study (NCGS), the Genetics and Neuroendocrinology of Short Stature International Study (GeNeSIS), and the Nordinet International Outcome Study (IOS) were previously the only other major databases for analysis of the effects of GH, but the value of these data was limited by the lack of accurate, objective adherence monitoring. KIGS was based on data from more than 60,000 patients in 50 countries, NCGS 47,000 patients in the USA and Canada, GeNeSIS 22,929 patients in 30 countries and Nordinet 17,995 patients in 19 countries. Self-reporting by patients, parents or caregivers, counting of prescriptions filled or vials of GH used were the only data sources available for tracking adherence in these databases and these are known to be unreliable [1, 7]. However, these large surveillance studies have established themselves as valuable primary sources of information about efficacy in a real-world setting, growth prediction modeling, treatment effects in rare conditions and adverse events during long-term GH treatment in children [32–35].

The rapid increase in the amount of biomedical RWD derived from tools and technologies like the easypod™ ecosystem has led to discussions on the best way to share the data and analyses derived from these ‘big data’. Open access online platforms such as www.sleepdata.org and the Big Data to Knowledge Initiative (https://commonfund.nih.gov/bd2k) are being created to address some of the challenges inherent in analysis of large, complex datasets and to stimulate data-driven discovery. The FAIR principles have been developed to ensure that these datasets are Findable, Accessible, Interoperable, and Reusable. The advantages of RWD include the accuracy and objectivity of data provided by reliable electronic devices and apps and the sheer scale of the datasets they can be used to develop. Machine learning and artificial intelligence (AI) are gradually evolving to cope with the scale and complexity of analyzing these large and heterogeneous datasets. The Internet of Things (IoT) suggests that the range of sources for RWD will increase rapidly in the near future as more electronically enhanced devices, such as smart watches, pill bottles, inhalers, insulin pens, continuous glucose monitors, ingestible sensors and even bathroom scales, become available. Potential weaknesses of RWD include the absence of individual clinical characteristics and background information, which is partially due to the challenges of integrating sensor/device data into EHRs [36].

There are potential ethical and legal aspects of collecting and analyzing large-scale datasets taken directly from patients’ devices or apps on smartphones. These include the issues around obtaining consent from the patients and the need to maintain privacy and confidentiality through anonymizing the individual data. The easypod™ ecosystem balances the need for HCPs to be able to access the adherence and other data from their individual patients with the ability to analyze large-scale datasets by anonymizing the data once they are collated into the database. The age, sex, stage of puberty (Tanner stage), rate of transmission of data from patient to HCP and details of time, date and dose administered are all that researchers can enter and obtain from the database. The rate of transmission of data is an alternative, less direct assessment of adherence. Patients choose how often they send their data using the transmitter and analysis shows that more frequent transmission correlates with higher adherence to the medication regimen. The results of any analysis of these data are highly aligned with real-world practice and can corroborate individual case studies of patients using the device, which show that direct monitoring of adherence with this digitally enhanced device can be used to improve motivation and patient management.

Electronic monitoring devices and apps offer patients, caregivers, and HCPs many advantages to help them to achieve the best possible therapeutic outcomes. The two case reports of children from Finland who have been treated with GH via the easypod™ device show the importance of ensuring good adherence to GH therapy and how this can be achieved through remote long-term monitoring via an electronically enabled device. The two case studies were chosen to illustrate the importance of adherence to GH therapy and could be utilized to illustrate this concept in a medical education setting. Both cases show how good growth outcomes can be achieved by following treatment as described. They also show how electronic adherence management can complement normal clinical follow-up by providing HCPs with reliable information about patient adherence between visits. The case studies show how interventions can be tailored to individual patients through day-to-day observation of adherence. They provide a valid starting point for a meaningful discussion on the most appropriate interventions to maintain optimal adherence and to gain the best outcomes for individual children with a growth disorder. They also highlight the importance of parental support for the child and HCP support for the family.

High adherence is important because timely and adequate treatment with GH enables children with GHD and non-GHD conditions, such as chronic renal failure, Turner syndrome and for children born SGA, to achieve an adult height close to or within the normal range.[37–39] Strengths of the use of case studies include their obvious real-world clinical relevance, while their weakness is in the specificity of the individual cases, which do not allow generalized extrapolation to other patients.

The ecosystem created by the device, the connect software and the database described here has many features that make sustained adherence to GH treatment attainable for young, adolescent and older teenage patients. The same principles could be applied in other therapeutic areas, once suitable mobile electronic devices and relevant apps are available. In the management of asthma, for example, electronic trackers attached to inhalers can help HCPs to assess the individual’s adherence to this form of treatment and to differentiate between genuinely severe asthma and poorly controlled asthma that is caused by suboptimal adherence to regular inhaled maintenance therapy. This in turn enables more accurate targeting of expensive injectable biologic treatments to the patients who actually need them, while poor adherence can be addressed by the HCP in face-to-face interactions with the patients who just need to take their inhaled medication more regularly, as prescribed. Similarly, in the management of growth disorders requiring GH treatment, effective monitoring of adherence can maximize the cost-effectiveness of treatment by optimizing growth outcomes such as final height and other clinical outcomes such as body composition, bone mineral density, cardiac function, lipid profile, and quality of life [7].

## Conclusions

The explosion in use of mobile phones and other mobile electronic devices in the past decade, such as the device used to generate RWD in ECOS, has the potential to reshape medicine through analysis of ‘big data’ collected directly from patients. RWE from observational studies, such as the case studies presented here, can also inform future medical practice in ways that traditional RCTs are unable to do. However, issues around privacy, consent and confidentiality, motivation to record additional outcomes data to complement data that is recorded automatically, such as injection data and dosing, all need to be addressed by researchers wishing to utilize these data. The digital ecosystem described herein shows how the three datasets it contains enable HCPs to access individual data to monitor adherence to treatment, to ensure optimal health outcomes, whilst preserving anonymity for the patients when the data are collated into a database for use by researchers to analyze.

## Data Availability

The analysis was performed from data from ECOS study, which is Merck KGaA sponsored phase IV clinical trial, data sharing should be done in accordance with the European Federation of Pharmaceutical Industries and Associations (EFPIA) and the Pharmaceutical Research and Manufacturers of America’s(PhRMA) Principles for Responsible Clinical Trial Data Sharing. Merck KGaA, Darmstadt, Germany, believes that as a biopharmaceutical company, the sharing of information related to company sponsored clinical trials is central to our mission. The sharing of clinical trial Information enables the medical and scientific community to further develop the medical and scientific knowledge base and permits the public to make informed healthcare decisions. However, because information from company sponsored clinical trials may include confidential personal information and proprietary company information, Merck must ensure that information is provided only in response to legitimate scientific and medical requests and that information disseminated outside of the company properly protects all confidential. All clinical trial information must further be provided only in accordance with applicable laws and codes. Merck will share anonymized patient level, and study level data and redacted clinical study reports from clinical trials in patients with qualified scientific and medical researchers, upon researcher request via the Merck website portal (https://www.merckgroup.com/en/research/our-approach-to-research-and development/healthcare/clinical-trials/commitment-responsible-data-sharing.html), as necessary for conducting legitimate research. More information for researchers can be found on this website. Evaluation of the Research Proposal, as well as the qualifications and experience of the Lead Researcher and Research Team, will be conducted by appropriate and qualified person or board. The researcher must enter into a Data Sharing Agreement (“DSA”) with Merck. The standard DSA which must be entered into is posted on Merck’s website. Merck uploads the data to Cloud-Based Data Sharing Analytical Solution where researchers can conduct the approved analysis. A request form is available on the Merck website portal (https://www.merckgroup.com/en/research/our-approach-to-research-anddevelopment/healthcare/clinical-trials/commitment-responsible-data-sharing.html). A point of contact for information regarding data requests is Sascha-Marc Seidl (Sascha-Marc.Seidl@merckgroup.com).

## Abbreviations

DAS: data analysis set
ECOS: easypod™ Connect Observational Study
EHR: electronic health record
GeNeSIS: genetics and neuroendocrinology of short stature international study
GH: growth hormone
GHD: GH deficiency
HCP: healthcare provider
HV: height velocity
IOS: Nordinet international outcome study
IoT: internet of things
KIGS: the Pfizer international growth database
NCGS: national cooperative growth study
PII: personal identifiable information
PSP: patient support program
RCT: randomized controlled trial
RWD: real-world data
RWE: real-world evidence
SDS: standard deviation score
SGA: small for gestational age
